# Parametric investigation and RSM optimization of DBD plasma methods (direct & indirect) for H_2_S conversion in the air

**DOI:** 10.1016/j.heliyon.2024.e29068

**Published:** 2024-04-10

**Authors:** Seyed Ali Razavi Rad, Mohammadreza Khani, Hadi Hatami, Mojtaba Shafiee, Babak Shokri

**Affiliations:** a Laser and Plasma Research Institute, Shahid Beheshti University, Tehran, Iran; b Department of Physics, Shahid Beheshti University, Tehran, Iran

**Keywords:** H_2_S conversion, Response surface methodology, Dielectric barrier discharge, Non-thermal plasma, Ozone, Process optimization

## Abstract

Hydrogen sulfide (H_2_S) is known as a harmful pollutant for the environment and human health, and its emission control is a high priority. Non-thermal plasma is an effective technology in this field. In this study, for the first time, the performance of direct and indirect H_2_S plasma conversion methods was compared, optimized, and modeled with the CCD method. H_2_S was diluted in zero air, and the study investigated the effect of discharge power, relative humidity, total flow rate, initial H_2_S concentration, and their interactions. ANOVA results showed that the models for H_2_S conversion efficiency and energy yield were significant and efficient. The direct method achieved a maximum conversion efficiency of 56 % and energy yield of 3.43 g/kWh, while the indirect method produced 68 % conversion efficiency and 1.59 g/kWh energy yield. According to the process optimization results, the direct conversion method is more optimal than the indirect conversion method due to the presence of active species and high-energy electrons in the plasma treatment, and it is a better choice if there are suitable working conditions.

## 1 Introduction

Hydrogen sulfide (H_2_S) is a highly toxic and irritating gas with a foul odor similar to rotten eggs in low concentrations. This inorganic sulfide is colorless, flammable, corrosive, and soluble. Its high solubility is due to its ability to react with water and form sulfide ions readily (${\text{HS}}^{-}$ and $S_{2}^{-}$). Although the odor of hydrogen sulfide can be detected at a very low concentration (less than 10 ppm), the sense of smell is lost in just a few minutes after exposure due to olfactory fatigue. This makes it impossible to sense the level of dangerous concentrations. Inhaling hydrogen sulfide at a few hundred ppm may result in acute poisoning [1,2].

H_2_S is a by-product of oil and gas production found in the gas or crude oil underground. Microbial decay of sulfur compounds and microbial reduction of sulfate, as well as geothermal steam, wood pulping, and various natural sources, can all produce H_2_S [3,4]. Furthermore, H_2_S is emitted when organic materials decay, like in sewage treatment plants, animal industries, and mushroom compost facilities. However, it has numerous uses in different industries, including producing metallic sulfides, phosphorus, and oil additives [2].

There is a growing concern that odor pollution is not only harmful to the environment but may also cause adverse effects on human health. Removing H_2_S is a high priority due to its negative health effects and potential for damaging equipment. Offensive odors emanating from wastewater collection and treatment systems, etc., have been a significant concern for its neighboring environments, and residents in contact with the system can use them to complain. Although various factors contribute to the odors produced at these facilities, this study specifically targets the investigation and mitigation of H_2_S emissions.

There are several traditional methods to control odor gas emissions into the atmosphere, such as biofiltration, absorption (wet scrubbing), adsorption, and incineration (thermal and catalytic) [[5], [6], [7], [8], [9], [10], [11], [12]]. Unfortunately, there are limitations to each technology that is used to remove odor gas. For instance, absorption and adsorption methods transfer odor-causing compounds from the gas phase to liquid or solid adsorbents during scrubbing. That's what causes it to form other pollution while resolving odor problems. Also, there are some problems with other methods, such as high energy consumption and unstable treatment efficiency. The economic and technical limitations of these methods are especially relevant for removing low-concentration odor gases. Plasma technology is also used for odor gas control [[13], [14], [15], [16]]. Non-thermal plasma (NTP) processing has unique advantages that make it useful for odor gas abatement. For example, the rapid reaction at ambient temperature under atmospheric pressure, ease of operation, achievement of high electron energies within a short residence time, etc. In the past decades, plasma has been widely considered for controlling and treating polluting gases such as acid compounds [[17], [18], [19], [20], [21]] and volatile organic compounds [[22], [23], [24], [25]]; such progress is very encouraging. Plasma catalysis is also used for H_2_S decomposition [[26], [27], [28]]. Although NTP technology has been used to remove H_2_S [[29], [30], [31], [32], [33]], no studies have specifically compared direct Air plasma treatment or using its by-product, ozone, for H_2_S removal. The main aim of this study is to compare these two methods of H_2_S conversion by influencing different parameters in the conversion process.

To understand the effect of process parameters on the desired response, most researchers rely on the traditional “one factor at a time” (OFAT) method, i.e., changing one parameter and keeping the others constant. This traditional approach has problems such as the high number of tests, especially if there are many variables (which increases the cost and time), and not considering simultaneous interactions between the studied parameters. To solve these problems, a design of experiment technique can be used, which reduces the number of experiments by examining all possible conditions, and eventually, the effect of parameters can be discussed from every aspect. Response surface methodology (RSM) is a mathematical method determining the relationship between one or more response variables with several independent variables under study [34]. Response surface designs aim to optimize the response (output variable) affected by several independent variables (input variables). In each test, changes in the input variables are made to determine the causes of changes in the response variable. An essential aspect of RSM is the design of experiments, commonly known as DOE. This strategy was originally developed for fitting experimental models but can also be used for numerical experiments. The DOE's purpose is to select the points at which the response should be evaluated. The choice of experimental designs can greatly impact the estimation accuracy and the cost of constructing the response surface model. In a traditional DOE, screening tests are performed early in the process when there are potentially many design variables that may have little or no effect on the response. In engineering sciences, many phenomena are modeled based on their theories, while many phenomena cannot have a suitable mathematical model due to many controlling factors, unknown mechanisms, or computational complexity; in such cases, experimental modeling methods are effective. So, the RSM is one of the methods of experimental modeling and research approaches in designing experiments and related sciences [35,36]. In RSM, an attempt is made to find a way to estimate interactions, quadratic effects, and even the local shape of the studied response surface by using a suitable experimental design. In other words, the RSM designs an empirical experiment that models the multiple effects of variables. Then, presenting a regression model establishes a relationship between the answer and the factors. In the meantime, specific goals are seriously pursued, the most important of which is to improve the process by finding optimal inputs, fixing the problems and weak points of the process, and stabilizing it. Other goals of this method include obtaining an optimal response curve for faster access, finding an indicator of the influence of the variables, and preparing a general report of the change process. Here, stabilization is an important concept in quality statistics that implies minimizing the effects of secondary or uncontrolled variables [37].

In this study, since it was necessary to use RSM to evaluate better the binary interactions of process parameters in the H_2_S conversion process and investigate the performance of NTP, the primary motivation underlying the present study was to develop a strategy by which the performance of NTP in H_2_S conversion using RSM, both directly and indirectly, be compared. The purpose of the comparison was to examine the impact of these parameters on both short-lived and long-lived plasma species and their performance on H_2_S conversion. At first, two distinct designs were employed. The first design was used to determine the maximum level of ozone saturation. Then, the range of power in which the ozone production rate was increasing and was not saturated was identified as the target range of one of the second design variables: the comparison of direct and indirect methods of H_2_S conversion. In this comparison, a dielectric barrier discharge (DBD) plasma reactor was used to investigate various parameters including relative humidity (RH), total flow rate, and H_2_S initial concentration.

## 2 Experimental

### 2.1 Experimental apparatus

**Fig. 1:**
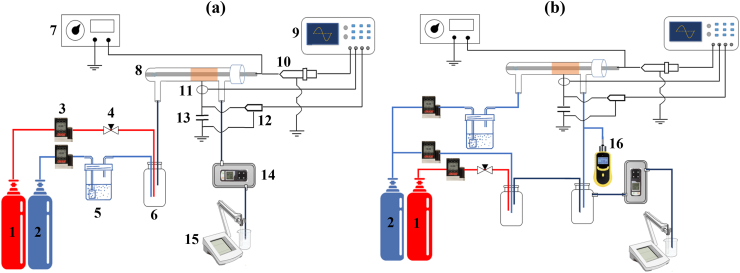
Schematic diagram of the experimental. (a) direct and (b) indirect setup. 1. Diluted H_2_S gas cylinder 2. Zero Air gas cylinder 3. MFC 4. Needle valve 5. Humidifier 6. Mixing chamber 7. AC power supply 8. DBD reactor 9. Oscillograph 10. High-voltage probe 11. Current probe 12. Voltage probe 13. Capacitor 14. H_2_S analyzer 15. pH meter 16. Ozone analyzer.

This work focused on atmospheric pressure NTP generation by dielectric barrier discharge (DBD) in a cylindrical reactor. As schematically shown in Fig. 1, the experimental setup includes two parts: (a) direct and (b) indirect. Each consists of a DBD reactor, a continuous flow gas supply system, electric and gaseous analytical systems, and an AC power supply (12 kV, 32 kHz, sine wave). The coaxial cylindrical DBD reactor was made of quartz glass with an inner diameter of 16 mm and wall thickness of 2 mm wrapped with a 50 mm long steel mesh, which acted as a ground electrode. The high-voltage electrode was a steel tube with an outer diameter of 12 mm placed on the axis of the reactor. In the direct method, diluted H_2_S was mixed with humidified air through a mixing chamber and then introduced into the DBD reactor (Fig. 1a) while, in the indirect method, H_2_S, after being diluted in the first mixing chamber reacted with the gas exiting the DBD reactor in the second mixing chamber (Fig. 1b). The relative humidity in the reactor was controlled at 5–65 % with a thermo-hygrometer. The flow rate and initial concentration of H_2_S were adjusted by mass flow controllers (MFC). The reactor temperature was also stabilized using a cooling fan. All experiments were performed after the temperature reached approximate stability.

### 2.2 Analyses and procedures

The range of Initial concentration of H_2_S was 56–136 $\text{mg}/m^{3}$ (determined by Senko SP12C7 Multi-Gas Detector) and the total flow rate in the reactor was in the range of 0.5–1.5 slpm.

The H_2_S removal efficiency is calculated by using equation (1):

(1) $${ɳ}_{H_{2}S}\left(\%\right)=\frac{C_{in}-C_{out}}{C_{in}}\times 100$$

where C_in_ and C_out_ represent the inlet and outlet concentrations of H_2_S ($\text{mg}/m^{3}$), respectively.

The concentration of ozone (O_3_) produced in the reactor was determined by an O_3_ analyzer (ATO-GAS_O3). Products of the reaction were detected by Fourier transform infrared (FTIR) analysis (Bruker Tensor 27 FTIR Spectrometer). During the experiment, the reaction products were added to distilled water to measure their pH by a pH/ORP analyzer (Milwaukee MW151 MAX pH/ORP/Temp Logging Bench Meter).

### 2.3 Electrical measurements

The generated plasma is atmospheric pressure plasma at room temperature. A high voltage probe (Tektronix P6015A), a current probe (Tektronix TCP202), a voltage probe (Tektronix P2220), and a 1 nF capacitance were placed in the circuit and connected to the oscilloscope (Tektronix DPO3012) to determine the instantaneous power and record the Lissajous figure.

The instantaneous power of plasma was monitored by oscilloscope according to equation (2):

(2) $$P_{P}\left(w\right)=V.I$$

where V and I are, respectively, the potential difference between the reactor electrodes and the electric current passing through the DBD reactor, which is measured by the high voltage and current probe.

The V-Q Lissajous diagram measured the electrical discharge power [38]. The electrical discharge power is directly proportional to the area of the parallelogram in the diagram and can be calculated by equation (3):

(3) $$P_{P}=f.C_{m}.A$$

where f is the frequency, C_m_ is the 1 nF measuring capacitance, and A is the area of the parallelogram.

To calculate the energy yield (EY) as a measure of energy efficiency, the specific input energy (SIE) was defined as the average power consumed in the discharge, divided by the total flow rate according to equation (4):

(4) $$SIE\left(J/l\right)=\frac{P_{P}\left(W\right)}{Q\left(l/\min\right)}\times 60$$

where P_P_ and Q represent the discharge power (W) and total flow rate (slpm).

Also, the energy yield for H_2_S conversion and O_3_ production was defined by equations (5), (6) respectively:

(5) $${EY}_{H_{2}S}\left(g/kWh\right)=\frac{{\left[C_{in}-C_{out}\right]}_{H_{2}S}}{SIE}\times 3.6$$

(6) $${EY}_{O_{3}}\left(g/kWh\right)=\frac{C_{O_{3}}}{SIE}\times 3.6$$

where C_in_, C_out,_ and C_O3_ denote the inlet and outlet concentrations ($\text{mg}/m^{3}$) of H_2_S and O_3_ concentration respectively.

In this study, two design (one with three and another with five parameters) with a three-level CCD design is used to investigate the effects of each independent parameter and their interactions on the H_2_S conversion process performance. In the first design, the discharge power (X_1_), relative humidity (X_2_), and total flow rate (X_3_) have been determined as the independent parameters and inputs for the O_3_ production process design. The discharge power (A), relative humidity (B), total flow rate (C), initial concentration (D), and conversion method (E) have been identified as the independent parameters and inputs for the H_2_S conversion process design. To complete the designs, the O_3_ production rate (Y_1_) and its energy yield (Y_2_) are identified as the responses of the first design, and H_2_S conversion efficiency (R_1_) and its energy yield (R_2_) for the second design. Each numerical independent parameter consists of three different levels, coded as −1 (low), 0 (center), and +1 (high), as shown in Table 1. The conversion method is a categorical parameter coded as −1 (Direct) and +1 (Indirect) in the second design.

**Table 1:** Independent parameters with coded and actual values in CCD.

Design	Independent parameters	Parameter type	Coded factors	Level and range		
				Low [−1]	Center [0]	High [+1]
O_3_ production process	Discharge power (W)	Numeric	X_1_	1	4	7
	Relative humidity (%)	Numeric	X_2_	5	35	65
	Total flow rate (l/min)	Numeric	X_3_	0.5	1	1.5
H_2_S conversion process	Discharge power (W)	Numeric	A	1	3	5
	Relative humidity (%)	Numeric	B	5	35	65
	Total flow rate (l/min)	Numeric	C	0.5	1	1.5
	Initial concentration (mg/m^3^)	Numeric	D	56	96	136
	Conversion method	Categoric	E	Direct	–	Indirect

The fitting quality and the significance of the designed model can be examined by the coefficient of determination (R^2^) and the F-value, which were analyzed by the analysis of variance (ANOVA). The interactions of the independent parameters were investigated by the response surfaces and contour plots based on the designed model [38].

## 3 Results and discussion

### 3.1 DoE analysis

#### 3.1.1 Regression models

Table 2 and Table 3 show the design of experiments for ozone saturation and H_2_S conversion, respectively, designed based on the CCF method. The total number of experimental runs required is 17 and 54, respectively, including three replicated runs using the parameters of the experiments at the center points.

**Table 2:** Actual response of ozone production and energy yield at experimental design points.

Run order	Independent variables (X)			Responses (Y)	
	$X_{1}$: Discharge power (W)	$X_{2}$: Relative humidity (%)	$X_{3}$: Total flow rate (slpm)	$Y_{1}$: Ozone production rate (mg/m^3^)	$Y_{2}$: Energy yield (g/kWh)
1	1	5	0.5	413.4	12.4
2^a^	4	35	1	854.6	12.82
3	4	65	1	670.5	10.06
4^b^	4	35	1	856.5	12.85
5	7	35	1	1109.4	9.51
6	7	5	0.5	1399.5	6
7	1	65	0.5	205.8	6.17
8	4	35	1.5	788.5	17.74
9	4	5	1	1059.6	15.89
10	1	5	1.5	297.9	26.81
11	7	65	1.5	817.3	10.51
12	1	65	1.5	158.8	14.29
13^c^	4	35	1	840.6	12.61
14	1	35	1	229.3	13.76
15	7	5	1.5	1383.8	17.79
16	7	65	0.5	899.7	3.86
17	4	35	0.5	889.9	6.67

^a–c^Replicated experimental runs (Run order: 2, 4, 13).

**Table 3:** Experimental design matrix and results of H_2_S conversion.

Run order	Independent variables (A-D)					Responses (R)	
	A: Discharge power (W)	B: Relative humidity (%)	C: Initial concentration (mg/m^3^)	D: Total flow rate (slpm)	E: Conversion method	$R_{1}$: $H_{2}S$ conversion (%)	$R_{2}$: Energy yield (g/kWh)
1	1	5	136	1.5	Direct	29	3.55
2	1	5	56	1.5	Direct	56	2.82
3	1	5	56	0.5	Direct	64	1.08
4	1	65	136	0.5	Indirect	9	0.37
5	1	5	136	0.5	Indirect	23	0.94
6	3	35	136	1	Indirect	28	0.76
7	1	35	96	1	Indirect	25	1.44
8	5	65	56	0.5	Direct	100	0.34
9	5	65	56	0.5	Indirect	68	0.23
10	5	5	136	0.5	Direct	94	0.77
11	1	65	56	1.5	Indirect	10	0.5
12	5	65	136	0.5	Indirect	55	0.45
13	3	35	96	0.5	Indirect	39	0.37
14	1	65	56	0.5	Direct	39	0.66
15	1	35	96	1	Direct	48	2.76
16	5	5	56	0.5	Direct	100	0.34
17	5	5	56	1.5	Indirect	80	0.81
18	3	35	56	1	Direct	78	0.87
19	5	35	96	1	Indirect	65	0.75
20	1	65	136	1.5	Indirect	5	0.61
21	3	35	56	1	Indirect	41	0.46
22	5	65	56	1.5	Direct	91	0.92
23	3	35	136	1	Direct	61	1.66
24	1	5	136	0.5	Direct	37	1.51
25	1	5	56	1.5	Indirect	34	1.71
26	5	65	136	1.5	Indirect	49	1.2
27	5	5	136	1.5	Indirect	67	1.64
28^d^	3	35	96	1	Indirect	36	0.69
29^a^	3	35	96	1	Direct	77	1.48
30^b^	3	35	96	1	Direct	76	1.46
31	5	65	136	0.5	Direct	77	0.63
32	3	65	96	1	Indirect	30	0.58
33	3	35	96	0.5	Direct	82	0.79
34	1	65	56	1.5	Direct	31	1.56
35	3	35	96	1.5	Indirect	33	0.95
36	1	65	136	1.5	Direct	13	1.59
37	5	5	56	1.5	Direct	96	0.97
38	3	35	96	1.5	Direct	72	2.07
39	1	5	136	1.5	Indirect	21	2.57
40	5	5	136	1.5	Direct	80	1.96
41	5	35	96	1	Direct	98	1.13
42	5	65	56	1.5	Indirect	63	0.64
43	1	5	56	0.5	Indirect	37	0.62
44	5	5	136	0.5	Indirect	72	0.59
45	5	5	56	0.5	Indirect	85	0.29
46^e^	3	35	96	1	Indirect	35	0.67
47^c^	3	35	96	1	Direct	75	1.44
48	3	5	96	1	Indirect	52	1
49	3	5	96	1	Direct	78	1.5
50	5	65	136	1.5	Direct	64	1.57
51^f^	3	35	96	1	Indirect	37	0.71
52	3	65	96	1	Direct	52	1
53	1	65	56	0.5	Indirect	12	0.2
54	1	65	136	0.5	Direct	17	0.69

^a–f^Replicated experimental runs (Run order for direct (a-c): 29, 30, 47, and indirect method (d-f): 28, 46, 51).

The ANOVA analysis determines the significance and adequacy of regression models. Table 4 shows the summary of the ANOVA results of responses based on the data in Tables (S1–S4) in Supporting information. The low p-values of all responses (<0.0001) with >95 % confidence level indicate that all models are significant. Also, the F-values for all responses were higher than the critical value (3.68, 3.44, 1.95, and 2.08 for Y_1_, Y_2_, R_1,_ and R_2_, respectively), which indicates that the regression models are statistically significant [36,39]. Therefore, it can be confirmed that most of the variations in responses can be explained by regression models. The value of R^2^ for each model is close to 1, which presents the agreement of the experimental data with the predicted results calculated by the regression models. For the stability and validity of the regression models, the difference between the predicted R^2^ and the adjusted R^2^ must be less than 0.2, which, in this work, these values are well fitted for all responses. These results show that all the regression models are statistically significant and adequate for predicting and optimizing the direct and indirect H_2_S conversion process.

**Table 4:** Summary of the ANOVA for each response of ozone saturation and H_2_S conversion designs.

Response	Model	F-value	*P*-value	$R^{2}$	Adjusted $R^{2}$	Predicted $R^{2}$	Model term with highest F-value	Model terms with p-value <0.05
$Y_{1}$: Ozone production	Quadratic	470.10	<0.0001	0.9983	0.9962	0.9723	$X_{1}$	$X_{1}$, $X_{2}$, $X_{3}$, $X_{1}X_{2}$, $X_{1}^{2}$
$Y_{2}$: Ozone production energy yield	Reduced quadratic	247.25	<0.0001	0.9960	0.9919	0.9723	$X_{3}$	$X_{1}$, $X_{2}$, $X_{3}$, $X_{1}X_{2}$, $X_{1}X_{3}$, $X_{2}X_{3}$, $X_{1}^{2}$
$R_{1}$: H_2_S conversion	Reduced quartic	414.80	<0.0001	0.9980	0.9956	0.9876	E	A, B, C, D, E, AB, AD, AE, CE, DE, $A^{2}$, $B^{2}$, $C^{2}$, ABC, ABE, ACE, BCE, $A^{2}B$, $A^{2}E$, ${\text{AB}}^{2}$, $B^{2}E$, $D^{2}E$, ABCE
$R_{2}$: H_2_S conversion energy yield	Reduced quartic	663.74	<0.0001	0.9992	0.9997	0.9893	D	A, B, C, D, E, AB, AC, AD, AE, BC, BD, CD, CE, DE, $A^{2}$, $B^{2}$, $C^{2}$, ABC, ABD, ABE, ACD, ACE, ADE, BCD, $A^{2}B$, $A^{2}C$, $A^{2}E$, $AB^{2}$, $B^{2}E$, $C^{2}E$, ABCD, ${A^{2}B}^{2}$, $A^{2}\text{CE}$, $A^{2}\text{DE}$, ${\text{AB}}^{2}E$

As shown in Table 4, the p-value of the model terms less than the significant level (0.05 in this work) is considered important for the process responses. For example, in ozone production, $x_{1}$, $x_{2}$, $x_{3}$, $x_{1}x_{2}$ and $x_{1}^{2}$ are significant terms but $x_{2}^{2}$, $x_{3}^{2}$, $x_{1}x_{3}$, and $x_{2}x_{3}$ are not significant; in the same way, $x_{1}$, $x_{2}$, $x_{3}$, $x_{1}x_{2}$, $x_{1}x_{3}$, $x_{2}x_{3}$, and $x_{1}^{2}$ are identified as important terms for ozone production energy yield, while $x_{2}^{2}$ is an insignificant term (Tables S1 and S2). The effect of each process parameter and their interactions on the process responses are derived from the regression model (Equations (7)–(10)). To better study the effects of different process parameters and their interaction on the desired responses, 3D response surface and predicted contour plots derived from regression equations will be analyzed. If there is a weak interaction between two parameters in the 3D response surface and projected contour plots, the fitted response surface will be in the shape of a plane, and the contour plot lines will be straight. In contrast, if these two parameters strongly interact, the contour plot lines will be curved. To understand this interaction, it is also possible to use the response gradient form concerning one of these parameters. If they have a significant interaction, the response gradient for one of the parameters can be significantly different when changing the other parameter.

According to equations (7), (8), the relationships between the process parameters and the output responses of ozone production and its energy yield were established by the quadratic and reduced quadratic models, respectively.

(7) $$Y_{1}:\text{Ozone}\;\text{production}\;\text{rate}\;\left(\text{mg}/m^{3}\right)=+845.03+430.45X_{1}\;-\;180.21X_{2}\;-\;36.2X_{3}\;-\;89.95X_{1}X_{2}+8.05X_{1}X_{3}+0.23X_{2}X_{3}\;-\;171.53X_{1}^{2}+24.17X_{2}^{2}\;-\;1.68X_{3}^{2}$$

(8) $$Y_{2}:\text{Ozone}\;\text{production}\;\text{energy}\;\text{yield}\;\left(g/\text{kWh}\right)=+12.51-\;2.58X_{1}\;-\;3.4X_{2}+5.2X_{3}+1.17X_{1}X_{2}-\;0.51X_{1}X_{3}-\;1.43X_{2}X_{3}\;-\;0.82X_{1}^{2}+0.52X_{2}^{2}$$

Also, the relationships between process variables and the output responses of H_2_S conversion and energy yield were established by reduced quartic models, as shown in equations (10), (9).

(9) $$R_{1}:H_{2}S\;\text{conversion}\;\left(\text{mg}/m^{3}\right)=+56.06+22.5A\;-12B-7.89C-3.22D-18.86E+1.81\text{AB}+0.12\text{AC}-0.69\text{AD}-1.06\text{AE}+0.12\text{BC}-\;0.06\text{BD}-0.56\text{BE}-0.37\text{CD}+2.28\text{CE}+1.11\text{DE}+3.12A^{2}-2.88B^{2}-3.88C^{2}-1.94\text{ABC}-1.19\text{ABE}-0.56\text{ACD}-1.25\text{ACE}+\text{BCE}+3.5A^{2}B+4.69A^{2}E+2.63{\text{AB}}^{2}+6.69B^{2}E-1.81D^{2}E+0.69\text{ABCE}$$

(10) $$R_{2}:H_{2}S\;\text{conversion}\;\text{energy}\;\text{yield}\;\left(g/\text{kWh}\right)=+1.06-\;0.58A\;-\;0.23B+0.27C+0.47D-\;0.38E+0.23\text{AB}+0.05\text{AC}-0.09\text{AD}+0.23\text{AE}\;-\;0.09\text{BC}-\;0.15\text{BD}+0.08\text{CD}-0.12\text{CE}-0.17\text{DE}+0.46A^{2}-\;0.04B^{2}-0.13C^{2}+0.04\text{ABC}+0.11\text{ABD}-0.02\text{ABE}+0.03\text{ACD}-0.02\text{ACE}+0.05\text{ADE}-0.04\text{BCD}-0.08A^{2}B-0.05A^{2}C-0.05A^{2}E+0.34{\text{AB}}^{2}+0.15B^{2}E+0.05C^{2}E+0.02\text{ABCD}-0.29A^{2}B^{2}+0.12A^{2}\text{CE}+0.08A^{2}\text{DE}-0.1{\text{AB}}^{2}E$$

#### 3.1.2 Effect of process parameters on ozone production rate and its energy yield

**Fig. 2:**
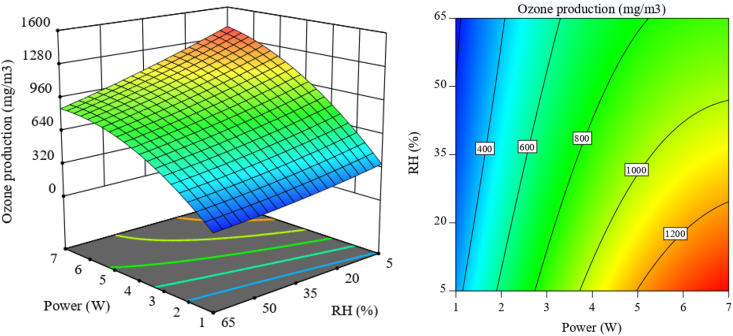
Ozone production rate as a function of discharge power and relative humidity with 1 slpm total flow rate ((a): 3D surface plot; (b): projected contour plot).

According to Fig. 2a, as expected, ozone production is suppressed by increasing humidity because increasing humidity increases breakdown voltage, which prevents the formation of a homogeneous plasma (the total flow rate was kept at 1 slpm) [40]. Moreover, it can be observed from the trend of changes in the graph that in the presence of humidity, with the increase of discharge power, the amount of ozone reaches saturation faster. The reason for this can be expressed that the presence of electronegative H_2_O molecules in the air gas flow quenches the high-speed electrons in the plasma and also changes the reaction pathway to O radical and O_3_ consumption (Equations (11)–(20)), which leads to a decrease in ozone concentration and earlier saturation [[41], [42], [43]]. Further, compared with the relative humidity, the discharge power has a more significant role in affecting O_3_ production, as evidenced by a larger lateral gradient in the 2D contour plot (Fig. 2b).

On the other hand, increasing the total flow rate, or in other words, reducing the gas residence time, has a much smaller effect than the relative humidity and discharge power on the ozone production rate and causes a slight decrease in it, which can be seen in Figure S1(a,b). The highest O_3_ production (∼1400 mg/m3) can be obtained with the highest discharge power of 7 W, the lowest total flow rate of 0.5 slpm, and the lowest relative humidity of 5 %.

(11) $$e+O_{2}\to O\left(3P\right)+O\left(3P,1D\right)+e$$

(12) $$O+O_{2}+O_{2}/N_{2}\to O_{3}+O_{2}/N_{2}$$

(13) $$H_{2}O+e↔\text{OH}+H+e$$

(14) $$O+H_{2}O\to \text{OH}+\text{OH}$$

(15) $$O+\text{OH}\to O_{2}+H$$

(16) $$O_{3}+\text{OH}\to O_{2}+HO_{2}$$

(17) $$O_{3}+H_{2}O\to H_{2}O_{2}+O_{2}$$

(18) $$HO_{2}+O_{3}\to \text{OH}+2O_{2}$$

(19) $$\text{OH}+\text{OH}\to H_{2}O_{2}$$

(20) $$H+HO_{2}\to H_{2}O_{2}$$

**Fig. 3:**
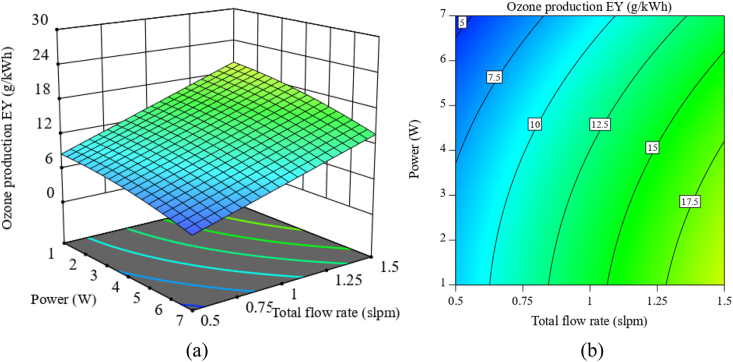
Ozone energy yield as a function of discharge power and total flow rate with 35 % RH ((a): 3D surface plot; (b): projected contour plot).

From Fig. 3a, it can be seen that reducing the discharge power and increasing the total flow rate has a beneficial effect on ozone production energy yield. To achieve the highest energy yield of O_3_ production (∼27 g/kWh), the discharge power and the relative humidity should be reduced to the lowest value of 1 W and 5 %, respectively; and the total flow rate to the highest value of 1.5 slpm. The relationship between energy yield and discharge power can be attributed to the fact that the increase in discharge power leads to a limited increase in micro-discharges. As the discharge power increases further, it causes an increase in current intensity and expansion of micro-discharges which results in energy loss in the form of heat [44]. Humidity also does not help to increase the energy yield because the increase in humidity reduces the ozone production rate and causes a decrease in the energy yield of ozone production (Figure S2a). Moreover, compared with the discharge power and relative humidity, the total flow rate is more effective for ozone energy yield, as evidenced in the 2D contour plot (Fig. 3b and Figure S2b).

As stated earlier, to better investigate the effect of the indirect method on H_2_S conversion, a range of discharge power in which the amount of ozone production has an increasing trend (1-5w) was considered for the discharge power variable in the second design.

#### 3.1.3 Effect of process parameters in H_2_S conversion

As summarized in Table 4, in the comparison of two direct and indirect methods for H_2_S conversion, A, B, C, D, E, AB, AD, AE, CE, DE, $A^{2}$, $B^{2}$, $C^{2}$, ABC, ABE, ACE, BCE, $A^{2}B$, $A^{2}E$, ${\text{AB}}^{2}$, $B^{2}E$, $D^{2}E$, and ABCE are identified as significant terms, while AC, BC, BD, BE, CD, and ACD play a weak role in the process, confirmed by the *P*-value (Table S3). Considering the highest F-value of 1699.68, it is believed that the conversion method is the most important parameter affecting the comparison of these two methods in H_2_S conversion.

**Fig. 4:**
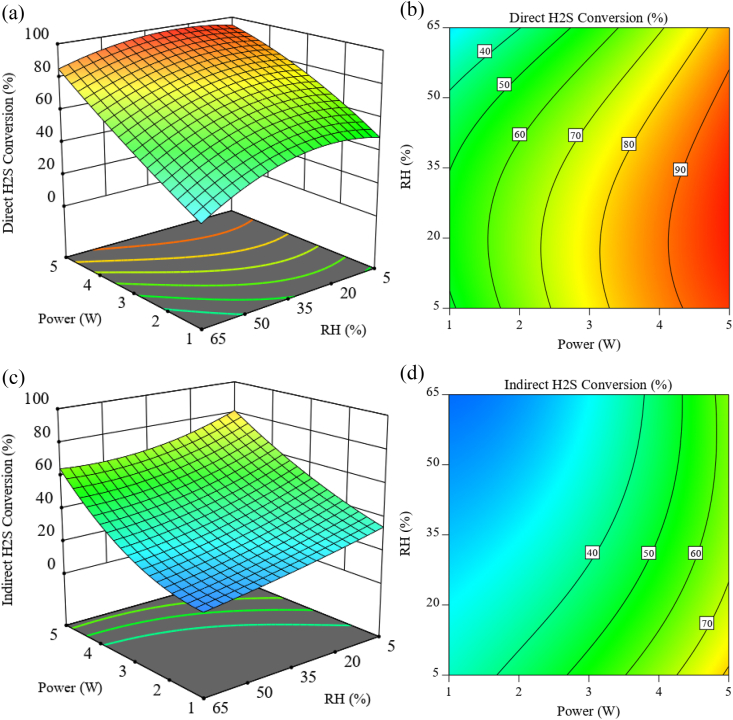
H_2_S conversion rate as a function of discharge power and relative humidity with 96 mg/m^3^ initial concentration and 1 slpm total flow rate ((a,b): 3D surface plot and projected contour plot of direct method; (c,d): 3D surface plot and projected contour plot of indirect method).

The combined effect of discharge power and relative humidity at an initial concentration of 96 mg/m^3^ and a total flow rate of 1 slpm on H_2_S conversion for direct and indirect methods, along with their contour plot, is presented in Fig. 4a. It can be stated that both in the direct and indirect methods, increasing the discharge power and decreasing the relative humidity has a positive effect on H_2_S conversion. However, comparing these two methods shows a difference in their plots related to relative humidity. In Fig. 4a, it can be seen that with the increase of humidity up to about 30 %, the conversion rate showed a relative increase and then decreased with the continuation of the humidity increase, while such changes are not observed in Fig. 4c. It seems that a low amount of relative humidity under plasma condition helps to more H_2_S conversion. This can be due to hydroxyl species (OH), which are very active species for chemical reactions. Still, when the humidity ratio in the reactor increases, because H_2_O molecules are more electronegative than H_2_S, in high humidity, they capture most of the electrons produced in the discharge, which reduces the number of electrons effective in the formation of electron avalanche and then leads to a decrease in conversion efficiency. Such a result has been seen similarly in other studies [27]. In the indirect method, OH species are decomposed before reaching the pollutant gas and do not play any role in the conversion. For the same reasons mentioned before, the conversion rate also decreases with the reduction of ozone production by humidity.

The mechanism of possible reactions in direct and indirect method, depending on free radicals and energetic atoms or electrons with and without humidity, is in the form of the equations in Table 5 and Table 6 (Equations (21), (22), (23), (24), (25), (26), (27), (28), (29), (30), (32), (33), (34), (35), (36), (37), (38), (39), (40), (41), (42), (43), (44), (46), (47), (48), (49), (50), (51), (52), (53), (54), (55), (56), (57), (58), (59), (60), (61), (62), (63)) [[45], [46], [47]]. In the direct method, there are two mechanisms for H_2_S conversion: (a) electron collision with H_2_S molecules and (b) reaction between O and OH radicals with H_2_S molecules [48]. Without humidity, equations (21), (23) are the dominant reactions for H_2_S conversion, and when H_2_O molecules are present, due to OH radicals, equations (21), (24) are considered the dominant reactions [46,48]. In the indirect method, whether in the presence of humidity or its absence, the dominant reaction for converting H_2_S is equation (33) [49]. However, in the presence of H_2_O, due to the consumption of O radicals and O_3_ molecules (equations (55), (57), (58)), the total concentration of O_3_ molecules decreases [49]. Therefore, since reaction (33) is the dominant pathway for the H_2_S conversion, the conversion efficiency decreases.

**Table 5:** The mechanism of possible reactions in direct and indirect method without humidity.

Without humidity		
Direct	Indirect	
(21) $H_{2}S+e\to \text{SH}+H$	a (31) $e+O_{2}\to O+O+e$	
a (22) $e+O_{2}\to O+O+e$	(32) $O+{2O}_{2}\to O_{3}+O_{2}$	
(23) $O+H_{2}S\to \text{SH}+\text{OH}$	(33) $H_{2}S+O_{3}\to SO_{2}+H_{2}O$	
(24) $\text{OH}+H_{2}S\to \text{SH}+H_{2}O$	(34) $H_{2}O+O_{3}+{\text{SO}}_{2}\to H_{2}SO_{4}$	
(25) $O_{2}+\text{SH}\to \text{SO}+\text{OH}$	(35) $H_{2}S+O_{3}\to O_{2}+H_{2}SO_{4}$	
(26) $$\text{SH}+\text{SH}\to H_{2}S+S$$		
(27) $$\text{SO}+O\to {\text{SO}}_{2}$$		
(28) $$\text{SO}+O_{2}\to {\text{SO}}_{2}+O$$		
(29) $$SO_{2}+O\to {\text{SO}}_{3}$$		
(30) $$SO_{3}+H_{2}O\to H_{2}SO_{4}$$		

a Replicated equations.

**Table 6:** The mechanism of possible reactions in direct and indirect method with humidity.

With humidity		
Direct	Indirect	
* (36) $e+O_{2}\to O+O+e$	* (52) $e+O_{2}\to O+O+e$	
^a^ (37) $H_{2}O+e↔\text{OH}+H+e$	(53) $O+{2O}_{2}\to O_{3}+O_{2}$	
^b^ (38) $O+H_{2}O\to \text{OH}+\text{OH}$	^a^ (54) $H_{2}O+e↔\text{OH}+H+e$	
^c^ (39) $\text{OH}+\text{OH}\to H_{2}O_{2}$	(55) $O+\text{OH}\to O_{2}+H$	
(40) $O+H_{2}S\to \text{SH}+\text{OH}$	^b^ (56) $O+H_{2}O\to \text{OH}+\text{OH}$	
(41) $\text{OH}+H_{2}S\to \text{SH}+H_{2}O$	(57) $O_{3}+H_{2}O\to H_{2}O_{2}+O_{2}$	
(42) $O_{2}+\text{SH}\to \text{SO}+\text{OH}$	(58) $O_{3}+\text{OH}\to O_{2}+HO_{2}$	
(43) O+SH→SO+H	(59) $HO_{2}+O_{3}\to \text{OH}+2O_{2}$	
(44) $\text{SO}+\text{OH}\to {\text{SO}}_{2}+H$	^c^ (60) $\text{OH}+\text{OH}\to H_{2}O_{2}$	
(46) $\text{OH}+\text{SH}\to S+H_{2}O$	(61) $H_{2}S+O_{3}\to SO_{2}+H_{2}O$	
(47) $S+O\to {\text{SO}}_{2}$	(62) $H_{2}O+O_{3}+{\text{SO}}_{2}\to H_{2}SO_{4}$	
(48) $H_{2}O+SO_{2}\to H_{2}SO_{3}$	(63) $H_{2}S+O_{3}\to O_{2}+H_{2}SO_{4}$	
(49) $$\text{OH}+SO_{2}\to {\text{HSO}}_{3}$$		
(50) $$H_{2}O+SO_{3}\to H_{2}SO_{4}$$		
(51) $${\text{HSO}}_{3}+\text{OH}\to H_{2}SO_{4}$$		

*, a, b, and c are replicated equations.

According to the 2D contour plots of these two methods, it can be seen that in both methods (Fig. 4b and d), the discharge power is more effective than the relative humidity.

**Fig. 5:**
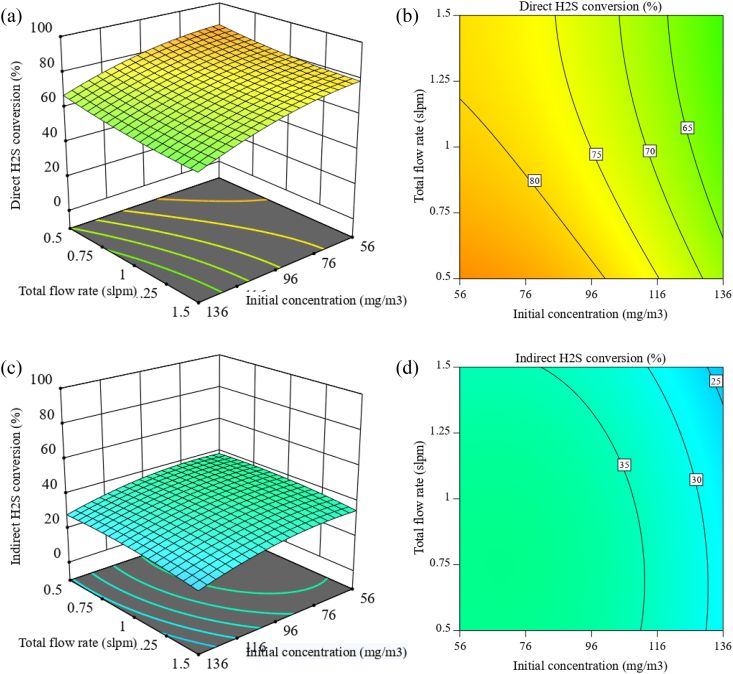
H_2_S conversion rate as a function of initial concentration and total flow rate with 3 W discharge power and 35 % RH ((a,b): 3D surface plot and projected contour plot of direct method; (c,d): 3D surface plot and projected contour plot of indirect method).

The effect of the initial concentration and total flow rate on the conversion rate in both methods is shown in Fig. 5a-d, while the discharge power and RH were kept at 3 W and 35 %, respectively.

In both methods, the initial concentration has a greater effect than the total flow rate, and the conversion rate increases as the initial concentration increases and the total flow rate decreases. The effect of the total flow rate is related to the gas residence time, which causes the effective species in chemical reactions to have more time to react. Regarding the reason for changes in the conversion rate due to changes in the initial concentration, it can be said that the conversion rate of H_2_S depends on the ratio of active species or energetic electrons to the number of H_2_S molecules [50]. At the same condition of the parameters, the energy density remains almost the same, and there are approximately no changes in the number and energy quantity of the active species; but when the initial concentration of H_2_S increases, the energy density of these species, which was almost constant, will no longer be sufficient to decompose the amount of added H_2_S molecules. As a result, the conversion rate of H_2_S decreases with increasing initial concentration.

#### 3.1.4 Effect of process parameters in H_2_S conversion energy yield

In comparing two direct and indirect methods for H_2_S conversion energy yield, as shown in Table 4, A, B, C, D, E, AB, AC, AD, AE, BC, BD, CD, CE, DE, $A^{2}$, $B^{2}$, $C^{2}$, ABC, ABD, ABE, ACD, ACE, ADE, BCD, $A^{2}B$, $A^{2}C$, $A^{2}E$, $AB^{2}$, $B^{2}E$, $C^{2}E$, ABCD, ${A^{2}B}^{2}$, $A^{2}\text{CE}$, $A^{2}\text{DE}$, and ${\text{AB}}^{2}E$ are important terms in the model of this response. The model has no insignificant term, which can be seen in the *P*-value column of Table S4. The total flow rate with the highest F-value of 6807.14 is the most important parameter influencing the comparison of these two methods in the H_2_S conversion energy yield.

**Fig. 6:**
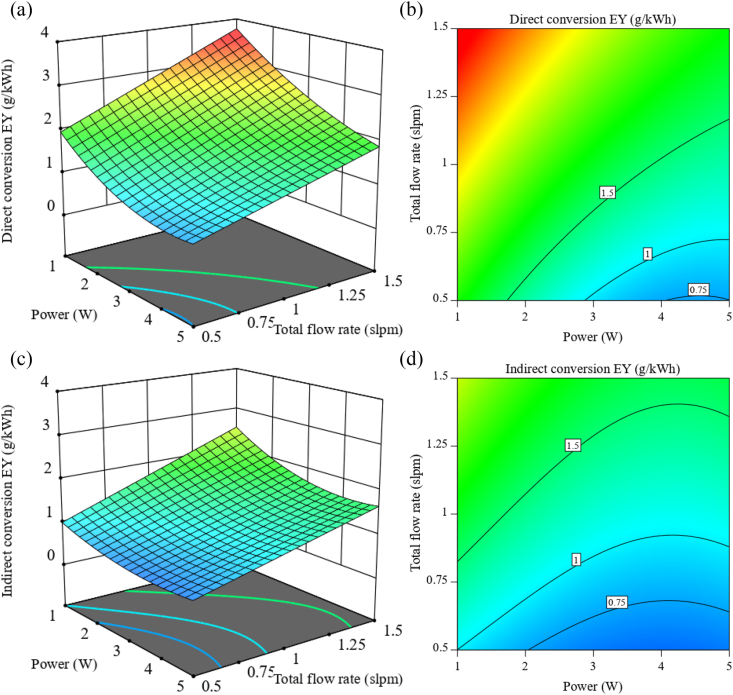
H_2_S conversion energy yield as a function of discharge power and total flow rate with 136 mg/kWh initial concentration ((a,b): 3D surface plot and projected contour plot of direct method with 20 % RH; (c,d): 3D surface plot and projected contour plot of indirect method with 5 % RH).

From Fig. 6a, it can be seen that to achieve the highest energy yield of H_2_S conversion in the direct method (∼3.7 g/kWh), the total flow rate and initial concentration should be increased to the highest values of 1.5 slpm and 136 mg/kWh, respectively; and the discharge power should be reduced to the lowest value of 1 W. Also, the relative humidity should be around 20 %. In the indirect method (Fig. 6c), changes in the total flow rate, initial concentration, and discharge power to reach the maximum conversion energy yield (∼2.5 g/kWh) are similar to the direct method. Still, the relative humidity is suggested to be reduced to its lowest value (5 %). This difference in the participation of RH can be related to the usefulness of having a low amount of RH for further H_2_S conversion in the direct method. Moreover, compared with the discharge power, the total flow rate is more effective for conversion energy yield in both methods, as evidenced in the 2D contour plot (Fig. 6b and d).

**Fig. 7:**
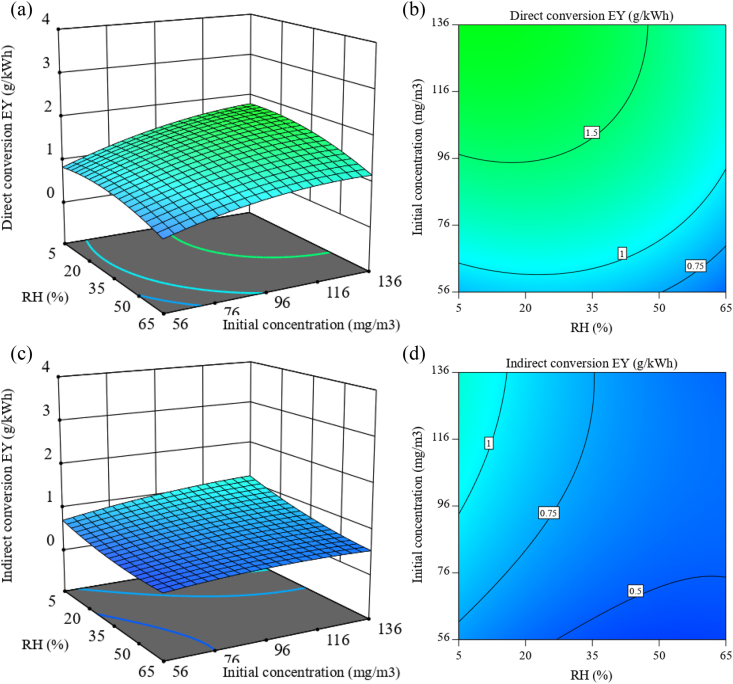
H_2_S conversion energy yield as a function of initial concentration and relative humidity with 3 W discharge power and 1 slpm total flow rate ((a,b): 3D surface plot and projected contour plot of direct method; (c,d): 3D surface plot and projected contour plot of indirect method).

As mentioned above, at a higher initial concentration, more H_2_S molecules are injected into the system. At the same time, the number of active species is insufficient to react with them, leading to a decrease in conversion efficiency. In Fig. 7a-d, the H_2_S conversion energy yield in both methods, defined as the ratio of the absolute amount of reduced H_2_S to the specific input energy (equation (5)), is plotted in terms of the initial concentration and relative humidity with 3 W discharge power and 1 slpm total flow rate. Higher initial concentration increases conversion energy yield despite conversion efficiency. This relation has been confirmed in many studies [17,[50], [51], [52]]. Based on this result, it can be stated that at a higher concentration of H_2_S molecules, the probability of encountering and interacting with active species increases, leading to more effective energy use.

Also, the effect of humidity on energy yield can be seen in Fig. 7. As shown earlier in Fig. 4, the increase in humidity causes a decrease in conversion efficiency because, with the entry of moisture, the probability of interaction of active species with H_2_S molecules decreases, thus ultimately leading to a decrease in energy yield. Moreover, according to the 2D contour plot of these two methods, it can be seen that in the direct method (Fig. 7b), the initial concentration is more effective than the relative humidity. In contrast, relative humidity is more important in the indirect method (Fig. 7d).

#### 3.1.5 Process optimization

The overall performance of plasma is highly dependent on operating conditions. Based on the obtained results, it can be concluded that the effects of the parameters on conversion efficiency and energy yield are different. For example, at a constant flow rate, higher discharge power increases H2S conversion efficiency but decreases process energy yield. However, at a higher flow rate, the process energy yield increases while the H_2_S conversion efficiency slightly decreases. Therefore, since achieving the highest values for both responses under the same operating conditions at the same time is impossible, it is necessary to optimize the H_2_S conversion process in two direct and indirect methods with multiple inputs and responses [53].

Here, process optimization aims to find a combination of parameters (inputs) that simultaneously maximize H_2_S conversion efficiency and energy yield (responses). For this purpose, the desirability function has been introduced as a suitable method to identify the possible points of the process parameters to obtain the optimal point of one or more responses [53,54]. In RSM, the desirability function is a mathematical tool used to evaluate the overall desirability of a set of responses based on their respective desirability values. The closer the desirability value is to 1, the closer the process is to the optimal point. However, it is important to note that individual desirability functions for different responses might be challenging to reconcile simultaneously, which can result in a lower overall desirability value [36].

Table 7 shows the optimal point obtained for the direct and indirect method of the H_2_S conversion process by process optimization along with the desirability value. The optimal point of the direct method (with a conversion efficiency of 56 % and energy yield of 3.426 g/kWh) is obtained at a discharge power of 1 W, relative humidity of 22 %, total flow rate of 1.5 slpm, and initial concentration of 73 mg/m^3^ with a desirability value of 0.72. In the same way, at a discharge power of 5 W, relative humidity of 5 %, total flow rate of 1.5 slpm, and initial concentration of 128 mg/m^3^, the optimal point of the indirect method (with conversion efficiency of 68 % and energy yield of 1.591 g/kWh) is introduced with a desirability value of 0.526. Finally, to compare this work with other works, the result of optimization along with some other studies was presented in Table 8, the results of our work were obtained without catalyst.

**Table 7:** Process optimization for the H_2_S conversion process.

Conversion method	Discharge power (W)	Relative humidity (%)	Total flow rate (slpm)	Initial concentration (mg/m^3^)	Conversion efficiency (%)	Energy yield (g/kWh)	Desirability
Direct	1	22	1.5	73	56	3.426	0.720
Indirect	5	5	1.5	128	68	1.591	0.526

**Table 8:** Comparison of the optimization results of this study with other works.

Ref.	SIE (J/l)	Carrier gas	Concentration (mg/m^3^)	Humidity (%)	Catalyst	Conversion (%)	Energy efficiency (g/kWh)
[55]	122.5	Air	30	30	Ceramic Raschig rings	93	0.61
[56]	225	Air	120	1.5	ACF	98	1.88
[57]	–	Air	75	0	ACF + TiO_2_/SMF	100	0.19
This work	(Direct) 40	Air	73	22	–	56	3.43
	(Indirect) 200	Air	128	5	–	68	1.59

### 3.2 By-products tracing

#### 3.2.1 IR spectra

**Fig. 8:**
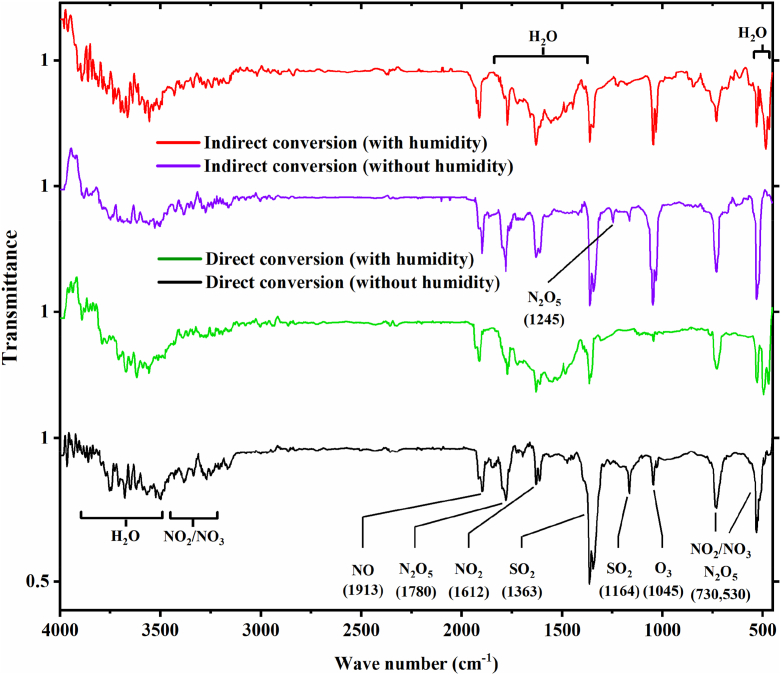
The results of IR absorption measurement of the direct and indirect method exhaust gases (with 50 % and without RH, 136 mg/m^3^ initial concentration, 1 slpm total flow rate, 3 W discharge power).

The direct and indirect method exhaust gases with and without humidity under the same condition (with 50 % or without RH, 3 W discharge power, 1 slpm total flow rate, and 136 mg/m^3^ initial concentration) were analyzed by FTIR with gas cell. Fig. 8 displays the Mid-infrared spectra (frequency range from 4000 to 400 cm^−1^) of the H_2_S conversion process by direct and indirect methods. The primary absorption lines of H_2_S have a central wavelength of 80 cm^−1^ [58]. Only a far-infrared spectrum with a wave number less than 400 cm^−1^ can measure H_2_S, so it doesn't display in this spectrum. As seen in Fig. 8, two of the four distinct regions for SO_2_ corresponding to different molecular vibrations of SO_2_ are evident according to studies [59,60]. The first region located at 500-600 cm^−1^ corresponds to the bending region ν_2_, the second region corresponds to the S=O symmetric stretching ν_1_, which is in the range of 1157–1172 cm^−1^ (1164 cm^−1^), and the third region corresponds to the asymmetric stretching ν_3_ in the range of 1321–1373 cm^−1^ (1363 cm^−1^). The last region, the combination region, is located at a wavelength of about 2500 cm^−1^ [59].

The SO_3_ molecule also has distinct regions but is less concentrated than SO_2_ [61]. On the other hand, at the wavelength of about 1386 cm^−1^, where the strongest absorption band of SO_3_ is located, it overlaps with the bands of SO_2_ and H_2_O, making it difficult to measure SO_3_ [61]. There is another band for SO_3_ at a wavelength of 2438 cm, which does not overlap with the H_2_O and SO_2_ bands and is separate from them, but this band is too weak to detect changes in SO_3_ concentration despite the noise in the absorption spectrum [61]. Other bands for SO_3_ can be considered (498 cm^−1^ and 530 cm^−1^), which also share IR absorption properties with H_2_O. The absorption peaks in the wavelength range of 1026–1064 cm^−1^ (1045 cm^−1^) can be attributed to the O_3_ molecule [62]. Fig. 8 also includes the other outlet species, such as NO, NO_2_, NO_3_, and N_2_O_5_ [62]. By analyzing the IR spectrum, differences between samples with and without humidity can be observed. In exhaust samples where the inlet gas was humid, less SO_2_ was absorbed. Based on this distinction, one can infer that when humidity is present, the reaction may create acidic compounds (such as H_2_SO_4_) [46]. It's also possible that the active species are more focused on interacting with H_2_O molecules rather than breaking down H_2_S. Either way, both outcomes ultimately lead to a decrease in SO_2_ production.

After examining both methods closely, it is noticeable that the peaks related to H_2_O molecules in the indirect method are more intense compared to the direct method but it is the opposite for SO_2_ peaks (in the direct method is more intense than in the indirect method). These differences can be attributed to the higher consumption of H_2_O molecules and sulfur atoms in the conversion of H_2_S to H_2_SO_4_ instead of the production of SO_2_ by short-lived species, as opposed to O_3_. Additionally, the intensity of the peaks related to nitrogen species is lower in the direct method compared to the indirect method. The reason behind this could be that in the indirect method, nitrogen species along with O_3_ were produced by the DBD reactor and then added to H_2_S molecules; While in the direct method, in addition to air molecules, H_2_S molecules are also introduced into the reactor, and with their involvement in the reaction, nitrogen species are produced less than before adding H_2_S.

Finally, it can be seen that the intensity of O_3_ molecules in the indirect method is higher compared to the direct method. This suggests that O_3_ is not completely consumed in the indirect method. On the other hand, in the direct method, the reaction proceeds less toward the production of O_3_ and focuses more on the interaction between short-lived species and H_2_S molecules.

#### 3.2.2 pH measurement

**Fig. 9:**
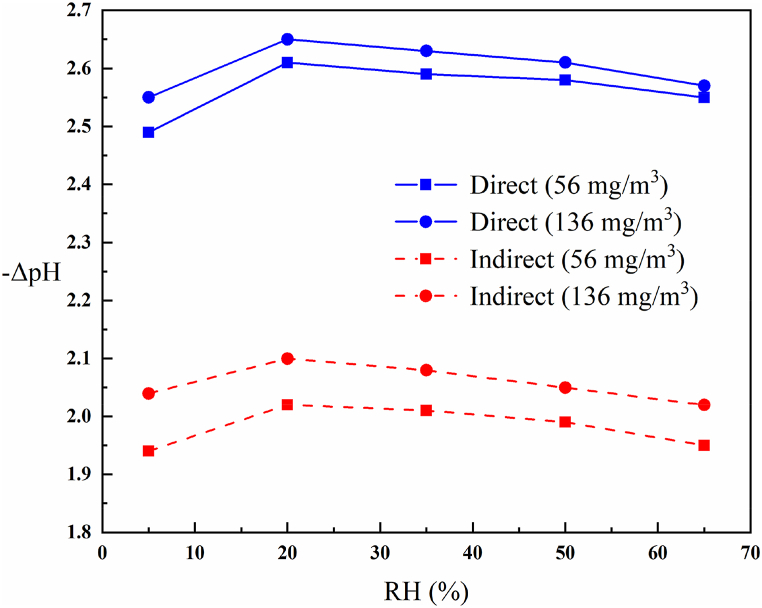
ΔpH as a function of RH in two direct and indirect methods with two initial concentrations of 56 and 136 mg/m^3^ (3 W discharge power, 1 slpm total flow rate).

The reactor's exhaust gas was added to distilled water to examine how humidity affects the formation of acidic species. The -ΔpH in terms of RH in two direct and indirect methods with two initial concentrations (56 and 136 mg/m^3^) is shown in Fig. 9.

As shown in Fig. 9, the reactor's exhaust gas either has acidic species with it or causes their formation in water because, in both methods, the pH of water shows its acidification. The direct conversion method significantly increases water acidity compared to the indirect conversion method, which can be related to its higher conversion efficiency. Also, the higher the initial concentration of H_2_S, the more acidic the water. This can also be related to more conversion of H_2_S. On the other hand, with increasing RH, it is observed that the water becomes more acidic. Still, with the continuation of the increasing process of RH, -ΔpH decreases slightly due to the less participation of H_2_S molecules in the reaction.

## 4 Conclusion

This study compared the performance of two H_2_S plasma conversion methods (direct and indirect conversion) using a DBD reactor, then optimized and modeled them with the CCD method. The ANOVA results showed that all models are significant and adequate. It was shown that in both methods, increasing the initial concentration of H_2_S and the total flow rate has a negative effect on the conversion efficiency and a positive effect on the energy yield. On the other hand, as expected, increasing the discharge power increases the conversion efficiency and decreases the energy yield. The effect of relative humidity was also investigated as another process parameter. It showed that in the direct conversion method, its low presence (30 %) leads to higher conversion efficiency due to the formation of OH radicals. Still, with its further increase, the conversion efficiency decreases. This initial increase in conversion efficiency was not seen in the indirect method, and the conversion efficiency started to decrease from the beginning of increasing RH. Another effect that RH had on the conversion process was that it caused the SO_2_ product to give way to H_2_SO_4_. Also, as long as RH positively affects H_2_S conversion efficiency, it also increases energy yield.

According to the process optimization results, the direct conversion method is more optimal than the indirect conversion method due to the presence of active species and high-energy electrons in the plasma treatment, and it is a better choice if there are suitable working conditions. The direct method achieved a maximum H_2_S conversion efficiency of 56 % and energy yield of 3.43 g/kWh, while the indirect method produced 68 % conversion efficiency and 1.59 g/kWh energy yield.

## CRediT authorship contribution statement

**Seyed Ali Razavi Rad:** Writing – original draft, Software, Methodology, Investigation, Formal analysis, Data curation. **Mohammadreza Khani:** Writing – review & editing, Visualization, Validation, Supervision, Resources, Project administration, Conceptualization. **Hadi Hatami:** Software, Methodology, Investigation, Formal analysis, Data curation. **Mojtaba Shafiee:** Software, Investigation, Formal analysis, Data curation. **Babak Shokri:** Writing – review & editing, Visualization, Validation, Resources.

## Declaration of competing interest

The authors declare that they have no known competing financial interests or personal relationships that could have appeared to influence the work reported in this paper.
