# Supplementary material for: Parametric investigation and RSM optimization of DBD plasma methods (direct & indirect) for H2S conversion in the air
Source: Heliyon. 2024 Apr 10;10(8):e29068. doi: 10.1016/j.heliyon.2024.e29068 (PMC11039977; doi:10.1016/j.heliyon.2024.e29068)
Supplement: Multimedia component 1 [file mmc1.docx]

### ANOVA for Quadratic model

**Response Y1: Ozone production rate**

Table S1. ANOVA table for ozone production rate quadratic model

| Source | Sum of Squares | df | Mean Square | F-value | p-value |  |
| --- | --- | --- | --- | --- | --- | --- |
| Model | 2.363E+06 | 9 | 2.626E+05 | 470.10 | < 0.0001 | significant |
| X_1_-Discharge power | 1.853E+06 | 1 | 1.853E+06 | 3317.44 | < 0.0001 |  |
| X_2_-Relative humidity | 3.248E+05 | 1 | 3.248E+05 | 581.45 | < 0.0001 |  |
| X_3_-Total flow rate | 13104.40 | 1 | 13104.40 | 23.46 | 0.0019 |  |
| X_1_X_2_ | 64728.02 | 1 | 64728.02 | 115.89 | < 0.0001 |  |
| X_1_X_3_ | 518.42 | 1 | 518.42 | 0.9282 | 0.3674 |  |
| X_2_X_3_ | 0.4050 | 1 | 0.4050 | 0.0007 | 0.9793 |  |
| X_1_² | 78831.76 | 1 | 78831.76 | 141.14 | < 0.0001 |  |
| X_2_² | 1564.97 | 1 | 1564.97 | 2.80 | 0.1381 |  |
| X_3_² | 7.58 | 1 | 7.58 | 0.0136 | 0.9105 |  |
| Residual | 3909.67 | 7 | 558.52 |  |  |  |
| Lack of Fit | 3758.86 | 5 | 751.77 | 9.97 | 0.0937 | not significant |
| Pure Error | 150.81 | 2 | 75.40 |  |  |  |
| Cor Total | 2.367E+06 | 16 |  |  |  |  |

Factor coding is **Coded**.
Sum of squares is **Type III - Partial**

The **Model F-value** of 470.10 implies the model is significant. There is only a 0.01% chance that an F-value this large could occur due to noise.

**P-values** less than 0.0500 indicate model terms are significant. In this case X_1_, X_2_, X_3_, X_1_X_2_, X_1_² are significant model terms. Values greater than 0.1000 indicate the model terms are not significant. If there are many insignificant model terms (not counting those required to support hierarchy), model reduction may improve your model.

The **Lack of Fit F-value** of 9.97 implies there is a 9.37% chance that a Lack of Fit F-value this large could occur due to noise. Lack of fit is bad -- we want the model to fit. This relatively low probability (<10%) is troubling.

### ANOVA for Reduced Quadratic model

**Response Y2: Ozone production EY**

Table S2. ANOVA table for ozone production EY quadratic model

| Source | Sum of Squares | df | Mean Square | F-value | p-value |  |
| --- | --- | --- | --- | --- | --- | --- |
| Model | 484.15 | 8 | 60.52 | 247.25 | < 0.0001 | significant |
| X_1_-Discharge power | 66.36 | 1 | 66.36 | 271.10 | < 0.0001 |  |
| X_2_-Relative humidity | 115.60 | 1 | 115.60 | 472.28 | < 0.0001 |  |
| X_3_-Total flow rate | 270.82 | 1 | 270.82 | 1106.41 | < 0.0001 |  |
| X_1_X_2_ | 10.88 | 1 | 10.88 | 44.45 | 0.0002 |  |
| X_1_X_3_ | 2.09 | 1 | 2.09 | 8.54 | 0.0192 |  |
| X_2_X_3_ | 16.33 | 1 | 16.33 | 66.72 | < 0.0001 |  |
| X_1_² | 2.04 | 1 | 2.04 | 8.32 | 0.0204 |  |
| X_2_² | 0.8183 | 1 | 0.8183 | 3.34 | 0.1049 |  |
| Residual | 1.96 | 8 | 0.2448 |  |  |  |
| Lack of Fit | 1.92 | 6 | 0.3207 | 18.75 | 0.0515 | not significant |
| Pure Error | 0.0342 | 2 | 0.0171 |  |  |  |
| Cor Total | 486.11 | 16 |  |  |  |  |

Factor coding is **Coded**.
Sum of squares is **Type III - Partial**

The **Model F-value** of 247.25 implies the model is significant. There is only a 0.01% chance that an F-value this large could occur due to noise.

**P-values** less than 0.0500 indicate model terms are significant. In this case X_1_, X_2_, X_3_, X_1_X_2_, X_1_X_3_, X_2_X_3_, X_1_² are significant model terms. Values greater than 0.1000 indicate the model terms are not significant. If there are many insignificant model terms (not counting those required to support hierarchy), model reduction may improve your model.

The **Lack of Fit F-value** of 18.75 implies there is a 5.15% chance that a Lack of Fit F-value this large could occur due to noise. Lack of fit is bad -- we want the model to fit. This relatively low probability (<10%) is troubling.

### ANOVA for Reduced Quartic model

**Response R1: H_2_S conversion**

Table S3. ANOVA table for reduced quartic model of H_2_S conversion

| Source | Sum of Squares | df | Mean Square | F-value | p-value |  |
| --- | --- | --- | --- | --- | --- | --- |
| Model | 38072.63 | 29 | 1312.85 | 414.80 | < 0.0001 | significant |
| A-Discharge power | 2025.00 | 1 | 2025.00 | 639.80 | < 0.0001 |  |
| B-Relative humidity | 576.00 | 1 | 576.00 | 181.99 | < 0.0001 |  |
| C-Initial concentration | 2240.44 | 1 | 2240.44 | 707.87 | < 0.0001 |  |
| D-Total flow rate | 373.78 | 1 | 373.78 | 118.10 | < 0.0001 |  |
| E-Conversion method | 5379.57 | 1 | 5379.57 | 1699.68 | < 0.0001 |  |
| AB | 105.13 | 1 | 105.13 | 33.21 | < 0.0001 |  |
| AC | 0.5000 | 1 | 0.5000 | 0.1580 | 0.6945 |  |
| AD | 15.13 | 1 | 15.13 | 4.78 | 0.0388 |  |
| AE | 40.11 | 1 | 40.11 | 12.67 | 0.0016 |  |
| BC | 0.5000 | 1 | 0.5000 | 0.1580 | 0.6945 |  |
| BD | 0.1250 | 1 | 0.1250 | 0.0395 | 0.8441 |  |
| BE | 11.11 | 1 | 11.11 | 3.51 | 0.0732 |  |
| CD | 4.50 | 1 | 4.50 | 1.42 | 0.2448 |  |
| CE | 186.78 | 1 | 186.78 | 59.01 | < 0.0001 |  |
| DE | 44.44 | 1 | 44.44 | 14.04 | 0.0010 |  |
| A² | 54.48 | 1 | 54.48 | 17.21 | 0.0004 |  |
| B² | 46.48 | 1 | 46.48 | 14.69 | 0.0008 |  |
| C² | 84.35 | 1 | 84.35 | 26.65 | < 0.0001 |  |
| ABC | 120.13 | 1 | 120.13 | 37.95 | < 0.0001 |  |
| ABE | 45.13 | 1 | 45.13 | 14.26 | 0.0009 |  |
| ACD | 10.13 | 1 | 10.13 | 3.20 | 0.0863 |  |
| ACE | 50.00 | 1 | 50.00 | 15.80 | 0.0006 |  |
| BCE | 32.00 | 1 | 32.00 | 10.11 | 0.0040 |  |
| A²B | 43.56 | 1 | 43.56 | 13.76 | 0.0011 |  |
| A²E | 123.20 | 1 | 123.20 | 38.93 | < 0.0001 |  |
| AB² | 24.50 | 1 | 24.50 | 7.74 | 0.0103 |  |
| B²E | 250.67 | 1 | 250.67 | 79.20 | < 0.0001 |  |
| D²E | 18.34 | 1 | 18.34 | 5.79 | 0.0241 |  |
| ABCE | 15.13 | 1 | 15.13 | 4.78 | 0.0388 |  |
| Residual | 75.96 | 24 | 3.17 |  |  |  |
| Lack of Fit | 71.96 | 20 | 3.60 | 3.60 | 0.1112 | not significant |
| Pure Error | 4.00 | 4 | 1.0000 |  |  |  |
| Cor Total | 38148.59 | 53 |  |  |  |  |

Factor coding is **Coded**.
Sum of squares is **Type III - Partial**

The **Model F-value** of 414.80 implies the model is significant. There is only a 0.01% chance that an F-value this large could occur due to noise.

**P-values** less than 0.0500 indicate model terms are significant. In this case A, B, C, D, E, AB, AD, AE, CE, DE, A², B², C², ABC, ABE, ACE, BCE, A²B, A²E, AB², B²E, D²E, ABCE are significant model terms. Values greater than 0.1000 indicate the model terms are not significant. If there are many insignificant model terms (not counting those required to support hierarchy), model reduction may improve your model.

The **Lack of Fit F-value** of 3.60 implies the Lack of Fit is not significant relative to the pure error. There is a 11.12% chance that a Lack of Fit F-value this large could occur due to noise. Non-significant lack of fit is good -- we want the model to fit.

### ANOVA for Reduced Quartic model

**Response R2: H_2_S conversion EY**

Table S4. ANOVA table for reduced quartic model of H_2_S conversion energy yield

| Source | Sum of Squares | df | Mean Square | F-value | p-value |  |
| --- | --- | --- | --- | --- | --- | --- |
| Model | 26.66 | 35 | 0.7617 | 663.74 | < 0.0001 | significant |
| A-Discharge power | 1.35 | 1 | 1.35 | 1172.51 | < 0.0001 |  |
| B-Relative humidity | 0.2116 | 1 | 0.2116 | 184.38 | < 0.0001 |  |
| C-Initial concentration | 0.2970 | 1 | 0.2970 | 258.82 | < 0.0001 |  |
| D-Total flow rate | 7.81 | 1 | 7.81 | 6807.14 | < 0.0001 |  |
| E-Conversion method | 2.19 | 1 | 2.19 | 1910.70 | < 0.0001 |  |
| AB | 1.63 | 1 | 1.63 | 1423.40 | < 0.0001 |  |
| AC | 0.0790 | 1 | 0.0790 | 68.84 | < 0.0001 |  |
| AD | 0.2398 | 1 | 0.2398 | 208.93 | < 0.0001 |  |
| AE | 0.2209 | 1 | 0.2209 | 192.48 | < 0.0001 |  |
| BC | 0.2503 | 1 | 0.2503 | 218.08 | < 0.0001 |  |
| BD | 0.7412 | 1 | 0.7412 | 645.82 | < 0.0001 |  |
| CD | 0.2064 | 1 | 0.2064 | 179.85 | < 0.0001 |  |
| CE | 0.0600 | 1 | 0.0600 | 52.30 | < 0.0001 |  |
| DE | 0.1225 | 1 | 0.1225 | 106.74 | < 0.0001 |  |
| A² | 0.5967 | 1 | 0.5967 | 519.95 | < 0.0001 |  |
| B² | 0.0053 | 1 | 0.0053 | 4.60 | 0.0458 |  |
| C² | 0.0450 | 1 | 0.0450 | 39.21 | < 0.0001 |  |
| ABC | 0.0428 | 1 | 0.0428 | 37.28 | < 0.0001 |  |
| ABD | 0.3720 | 1 | 0.3720 | 324.11 | < 0.0001 |  |
| ABE | 0.0088 | 1 | 0.0088 | 7.65 | 0.0127 |  |
| ACD | 0.0319 | 1 | 0.0319 | 27.78 | < 0.0001 |  |
| ACE | 0.0149 | 1 | 0.0149 | 12.96 | 0.0020 |  |
| ADE | 0.0914 | 1 | 0.0914 | 79.62 | < 0.0001 |  |
| BCD | 0.0520 | 1 | 0.0520 | 45.31 | < 0.0001 |  |
| A²B | 0.0244 | 1 | 0.0244 | 21.25 | 0.0002 |  |
| A²C | 0.0109 | 1 | 0.0109 | 9.48 | 0.0065 |  |
| A²E | 0.0137 | 1 | 0.0137 | 11.91 | 0.0028 |  |
| AB² | 0.4148 | 1 | 0.4148 | 361.45 | < 0.0001 |  |
| B²E | 0.1187 | 1 | 0.1187 | 103.44 | < 0.0001 |  |
| C²E | 0.0130 | 1 | 0.0130 | 11.29 | 0.0035 |  |
| ABCD | 0.0081 | 1 | 0.0081 | 7.08 | 0.0159 |  |
| A²B² | 0.0657 | 1 | 0.0657 | 57.29 | < 0.0001 |  |
| A²CE | 0.0478 | 1 | 0.0478 | 41.64 | < 0.0001 |  |
| A²DE | 0.0248 | 1 | 0.0248 | 21.57 | 0.0002 |  |
| AB²E | 0.0362 | 1 | 0.0362 | 31.57 | < 0.0001 |  |
| Residual | 0.0207 | 18 | 0.0011 |  |  |  |
| Lack of Fit | 0.0191 | 14 | 0.0014 | 3.40 | 0.1229 | not significant |
| Pure Error | 0.0016 | 4 | 0.0004 |  |  |  |
| Cor Total | 26.68 | 53 |  |  |  |  |

Factor coding is **Coded**.
Sum of squares is **Type III - Partial**

The **Model F-value** of 663.74 implies the model is significant. There is only a 0.01% chance that an F-value this large could occur due to noise.

**P-values** less than 0.0500 indicate model terms are significant. In this case A, B, C, D, E, AB, AC, AD, AE, BC, BD, CD, CE, DE, A², B², C², ABC, ABD, ABE, ACD, ACE, ADE, BCD, A²B, A²C, A²E, AB², B²E, C²E, ABCD, A²B², A²CE, A²DE, AB²E are significant model terms. Values greater than 0.1000 indicate the model terms are not significant. If there are many insignificant model terms (not counting those required to support hierarchy), model reduction may improve your model.

The **Lack of Fit F-value** of 3.40 implies the Lack of Fit is not significant relative to the pure error. There is a 12.29% chance that a Lack of Fit F-value this large could occur due to noise. Non-significant lack of fit is good -- we want the model to fit.

Figure S2. Ozone energy yield as a function of discharge power and relative humidity with 1 slpm total flow rate ((a): 3D surface plot; (b): projected contour plot).


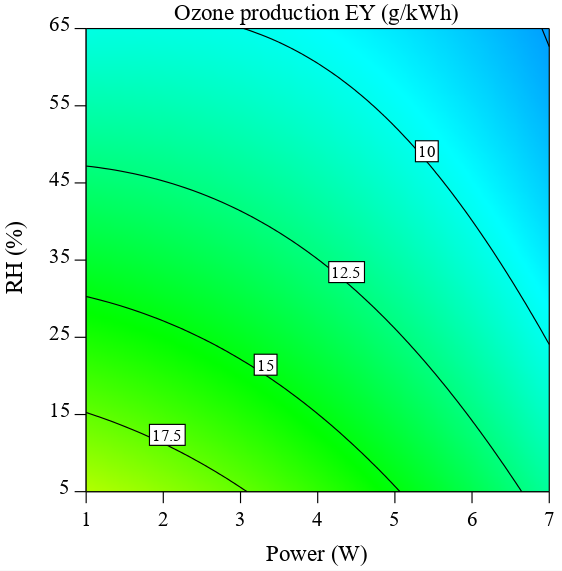

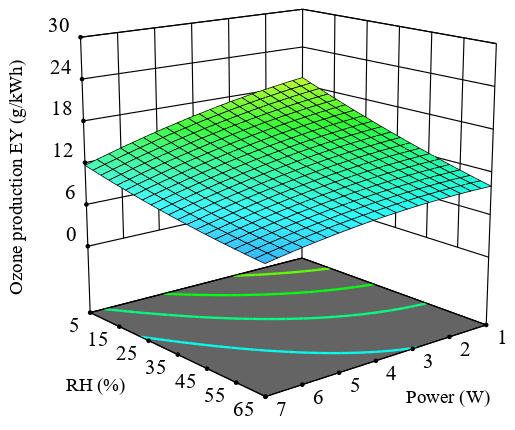


(a)

(b)


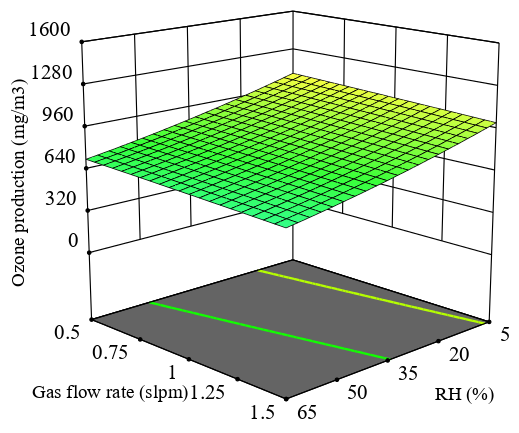

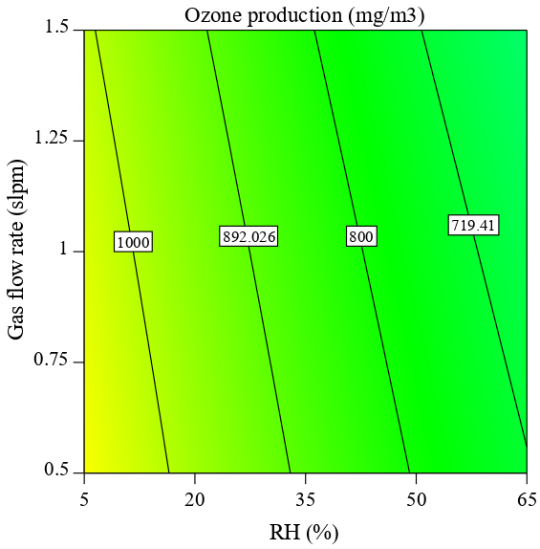


(a)

(b)

Figure S1. Ozone production rate as a function of total flow rate and relative humidity with 4 W discharge power ((a): 3D surface plot; (b): projected contour plot).
